# Open-source 3-D printable autoinjector: Design, testing, and regulatory limitations

**DOI:** 10.1371/journal.pone.0288696

**Published:** 2023-07-14

**Authors:** Anjutha Selvaraj, Apoorv Kulkarni, J. M. Pearce

**Affiliations:** 1 Faculty of Science, Medical Sciences and Environmental Sciences, Western University, London, ON, Canada; 2 Department of Electrical & Computer Engineering, Western University, London, ON, Canada; 3 Ivey Business School, Western University, London, ON, Canada; Mustansiriyah University, IRAQ

## Abstract

Autoinjectors have become popular modern injectable medical devices used as drug delivery systems. Due to their ease, capability and reliability compared to other conventional injectable medical devices, the market and manufacturing demand for autoinjector devices are increasing rapidly and expected to reach a market of $37.5 billion globally by 2025. Although autoinjectors can offset healthcare treatment costs through self-administered medication, they can be expensive for consumers, which limit their accessibility. This study describes the design and manufacture of a spring-driven and 3-D printed autoinjector to overcome this economic accessibility challenge. The digitally replicable device is released as open-source hardware to enable low-cost distributed manufacturing. The bill of materials and assembly instructions are detailed, and the effectiveness of the autoinjector is tested against the current standard (ISO 11608–1:2022) for needle-based injection systems. The safety and dosing accuracy was tested by measuring the weight of 100% ethyl alcohol expelled from six BD Insulin syringes with varying capacities or needle lengths. A one-way analysis assessed the variability between the dose delivery efficiency of 1mL, 0.5mL, and 0.3mL syringes. Testing indicated that the entire dose was delivered over 97.5% of the time for 1mL and 0.5mL syringes, but the autoinjector’s loaded spring force and size exceeded structural limitations of 0.3mL or smaller syringes. Components can be manufactured in about twelve hours using an open-source desktop RepRap-class fused filament 3-D printer. The construction requires two compression springs and 3-D printed parts. The total material cost of CAD$6.83 is less than a tenth of comparable commercial autoinjectors, which makes this approach promising. The autoinjector, however, is a class two medical device and must be approved by regulators. Future work is needed to make distributed manufacturing of such medical devices feasible and reliable to support individuals burdened by healthcare costs.

## 1. Introduction

Making the original invention of insulin effectively open-source was regarded as so crucial that Frederick Banting refused to put his name on the patent after discovering it [1]. The patent was later sold for $1 to the University of Toronto in an attempt to make it widely available. It was hypothesized long before the insulin breakthrough that the pancreas stored a substance that regulated the digestion of carbohydrates, and Frederick Banting, an orthopedic surgeon, had the idea of producing intravenous injections with the pancreatic extract of the dog; later, the injection was named Isletin [2]. The invention of insulin occurred before the advent of open-source philosophy [3, 4] and its application to discoveries such as hardware and drugs [5–7]. Thus, the generic production of insulin has yet to materialize, and there are significant cost problems [8]. Developments in the production of the drug, in tandem with advancements in the method of delivery following the first substantial use of insulin in 1921 to cure diseases at Toronto General Hospital [9], have kept prices high [10]. In many countries, there is wide use of insulin pens for administrating insulin, which widens the scope of delivery devices other than syringes in the medical and health sector [11]. The growth of delivery devices in the medical industry is taking place at a rapid pace, and the demand for devices such as syringes, autoinjectors, needle-free injectors, and insulin pumps is increasing due to the increasing rate of diseases [9, 12].

Autoinjectors are preferable over syringes and other conventional injectable devices where higher viscosities and larger volumes are needed [12]. Globally, autoinjectors are devices used by many healthcare practitioners, patients, and parents (for children under 12), to inject insulin into the body of diabetic individuals. Other chronic conditions, such as psoriasis, multiple sclerosis, and rheumatoid arthritis, can also be treated using an autoinjector. In addition, the device is essential during emergency conditions for migraine, anaphylaxis, and status epilepticus patients. With an estimated market valuation of $37.5 billion globally by 2025, the growth of injectable medical devices is increasing dramatically, compared to $16.7 billion in 2016 [13, 14]. The anticipated distribution of autoinjectors in 2025 is 9% of the total injectable drug delivery market share, compared to 6% in 2016 [14]. The anticipated distribution of other injectable devices like needle-free injectors, prefilled needles and pen injectors are 3%, 24%, and 64% in 2025 compared to 2%, 23%, and 69% in 2016 [14]. Autoinjectors are more reliable and easier to operate for self-administering to avoid patients making a hospital trip for insulin delivery. Various versions of autoinjectors can be purchased, varying in design, volume capacity, size, and degree of autonomy [15].

Autoinjectors are one of the most popular medical devices used to administer insulin into the bloodstream. Autoinjectors are, however, relatively expensive because of the complicated mechanical design compared to conventional injectable devices like syringes [16]. The administration of insulin with an autoinjector is not the only drug with a similar cost problem. In fact, auto-injection of epinephrine has caused a crisis with the EpiPen [14]. The EpiPen had a surge in price escalation when in nine years, Mylan, the drug manufacturer company, increased the price of a two-pack injectable epinephrine from $94 to $600 [17]. This staggering rise in price is due to the intellectual monopoly-based [18] patent system, which lays incentives for companies to innovate by allocating them to a 20-year exclusive monopoly [17]. According to Mylan, the increase in the cost to customers is due to the high deductible insurance plans in the market [17]. Thus, companies increase prices rapidly to earn more profit at the expense of patients and particularly those without insurance.

In many cases, hospital injections constitute almost half of the total treatment cost, which is why single-spring-actuated autoinjectors are cost-effective medical devices [19]. Additionally, the traction for autoinjectors has increased because of dosage accuracy, easy self-administration, improved patient compliance, and reduced anxiety [15]. Commercial autoinjectors, however, are relatively expensive due to their complex mechanical designs and the growing market value [13, 16]. The availability of a low-cost autoinjector would thus also have a social impact as it would directly improve the UN’s Sustainable Developmental Goal (SDG) 3 (Good health and well-being) while also making contributions to SDG1 (No Poverty) and SDG9 (Industry, Innovation and Infrastructure) [20].

One approach to reigning in medical costs and overcoming the drawbacks of the intellectual monopoly is to develop open-source hardware for medicine [21–24]. Therefore, investments in open hardware for medicine have the potential to reign in medical costs and overcome the drawbacks of intellectual monopoly [23]. This is possible because of the recent rapid development of distributed digital manufacturing techniques such as additive manufacturing or 3-D printing [25–28]. Combining an open-source approach and the use of distributed additive manufacturing would enable an autoinjector to fit the market in low-resource countries, which have a high prevalence of diseases such as diabetes [29]. To enable this socially-beneficial technical opportunity, this study aims to use the open hardware approach for medical hardware to enable the design and distributed manufacturing of cost-effective autoinjectors. This work includes designing and manufacturing of a spring-driven autoinjector in the open-source toolchain of FreeCAD, followed by slicing in open-source Cura and printing on an open-source RepRap class 3-D printer.

The bill of materials (BOM), assembly instructions, and design files [30] are released under an open-source license to ensure anyone may replicate, redesign, manufacture, or use the autoinjector. The device was tested by measuring the dose delivery efficiency for 1 mL, 0.5 mL, and 0.3 mL syringes in accordance with ISO 11608–1:2022 [31]. To ensure the autoinjector can perform as designed, 100% ethyl alcohol was used as a test liquid to mimic insulin, because they both have the viscosity of approximately 1.1 mPa⋅s at 20°C [32, 33]. Free-fall testing was also conducted on a 3mm thick surface of steel to test the durability of the device in the vertical and horizontal orientations.

## 2. Materials and methods

### 2.1. Design

**Fig 1:**
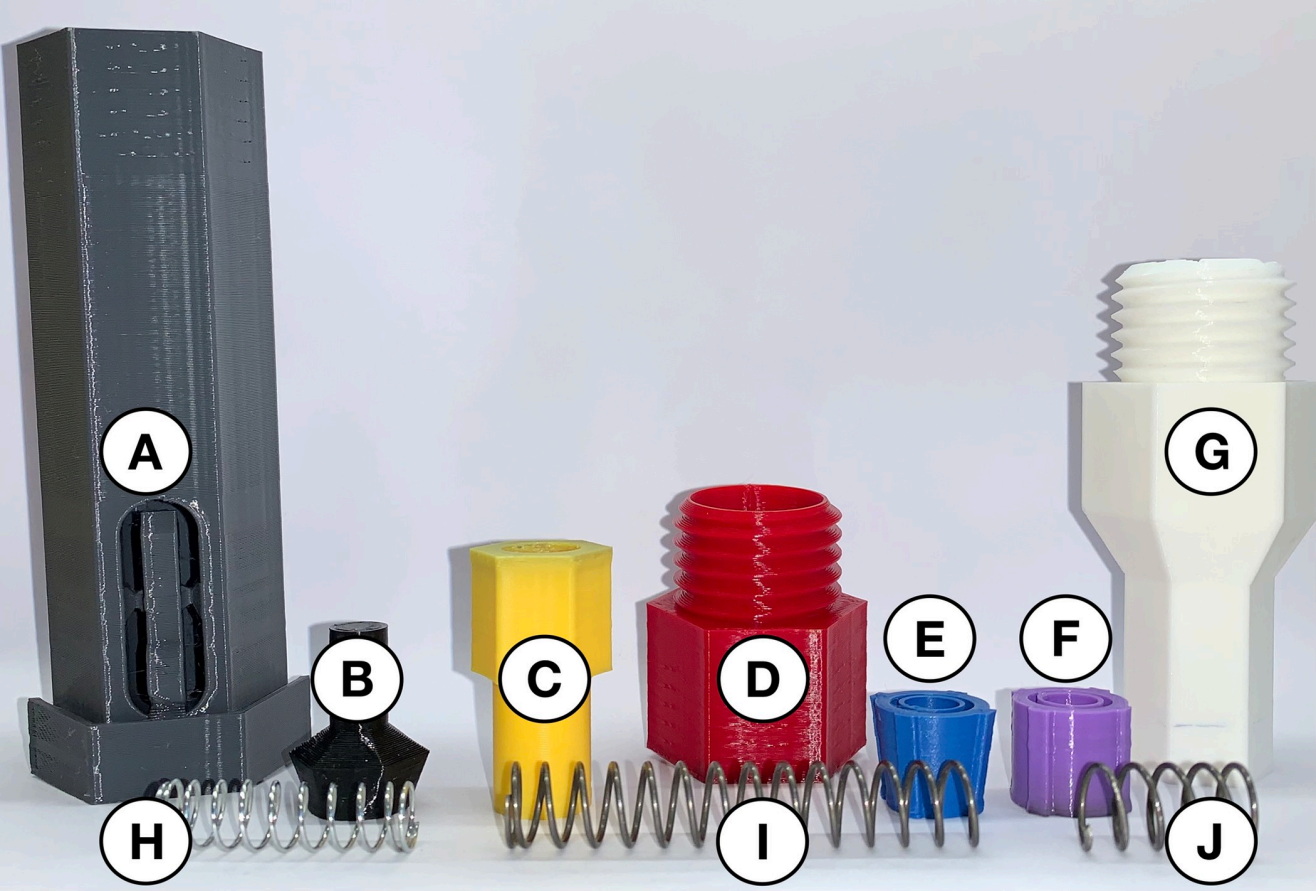
A visual guide to the open-source autoinjector’s digitally manufactured (parts *A*-*G*) and purchased components (parts *H*, *I*, and *J*). (*A*) Grey coloured injector cover is the first part placed in the back from left to right. (*B*) Black coloured two-spring-hold at the second position of the back row. (*C*) Yellow coloured plunger shown as the third part. (*D*) Red coloured connector in the centre of the back row. (*E*) Blue coloured spring-cone-retainer at the fifth position in the back row. (*F*) Purple coloured spring-retainer in the sixth position. (*G*) White coloured syringe-cover in the seventh position of the back row. (*H*) 35 mm compression spring in the front left corner of image. (*I*) 61.5 mm fraction of the 81.5 mm compression spring in the front centre. (*J*) 20 mm fraction of the 81.5 mm compression spring in the front right corner of image.

The open-source autoinjector is a spring-driven medical device designed to aid the self-administration of medication. The goal of this design is to make a low-cost device, which adheres to current ISO standards [31] and can be manufactured using a low-cost readily accessible 3-D printer and widely available materials. Open-source technology was used for 3-D modelling (FreeCAD 0.19 [33]), slicing (Ultimaker Cura 4.13.1 [34]), and printing (Creality Ender-3 [35]) this device. The source code (in FreeCAD FCStd file type) is made available in the design file repository on the Open Science Framework [29] for ease of customization or alterations. Additionally, STL files for each part are compatible with direct printing using any self-replicating rapid prototyper (RepRap)-class fused filament fabrication (FFF) printer [36–38]. To further improve the accessibility of this device, all 3-D-printed parts used polylactic acid (PLA) filament. This thermoplastic polymer is lightweight, common, and cost-effective. The design incorporates two springs and seven 3-D-printed parts, which are printed in different colors here to make the assembly instructions more accessible (Fig 1).

#### 2.1.1. Bill of materials

The BOM for the seven 3-D printed components and two springs is shown in Table 1. The device costs CAD$6.83, and the 3-D printed components can be manufactured in 12 hours and 6 minutes using the settings shown in Table 2. All components in the design are printed using generic PLA [39], which costs around CAD$25.95/kg. It should be noted that PLA is a biocompatible polymer [40]. The total weight of the 3-D printed components of the autoinjector is 108 g. Thus, the total cost of the 3-D printed parts is CAD$2.82. The seven components can be reproduced on any FFF printer with a 0.4 mm nozzle and the included print profile (OS AI 0.2mm.curaprofile [29]). Using this style of 3-D printer, the tolerances should be within 100 microns, which is the general resolution for these printers. External supports are not required for any of the components.

**Table 1:** Bill of materials for the device’s 3-D printed components and springs.

Part	Component	Material	Print Time	Mass (g)	Cost (CAD$)	Source
*A*	Injector Cover	PLA	5h 37min	55	1.43	[29]
*B*	Two-Spring-Hold	PLA	25min	3	0.08	[29]
*C*	Plunger	PLA	40min	6	0.16	[29]
*D*	Connector	PLA	1h 44min	11	0.29	[29]
*E*	Spring-Cone-Retainer	PLA	16min	2	0.05	[29]
*F*	Spring-Retainer	PLA	19min	3	0.08	[29]
*G*	Syringe-Cover	PLA	3h 5min	28	0.73	[29]
*H*	35 mm Compression Spring	Zinc Plated Steel	-	0.8	0.12	[42]
*I/J*	81.5 mm Compression Spring	Music-Wire Steel	-	3.81	3.89	[41]

**Table 2:** Print settings for 3-D printed components of autoinjector.

Part	Layer Height	Infill	Printing Temperature	Build Plate Temperature	Print, Wall, and Top/ Bottom Speed	Initial Speed
*A-G*	0.2 mm	75% Triangle pattern	207°C	65°C	75 mm/s	50 mm/s 2 layers

The total cost of the autoinjector was $6.83, and the 81.5 mm compression spring [41] was the most expensive component at $3.89. Many commercial devices use multiple springs of varying dimensions and stiffness. That was avoided, however, when designing the autoinjector to increase the accessibility of this device further. By using wire cutters to create two spring components from the 81.5 mm compression spring (parts *I* and *J*), the cost of the device remained low.

### 2.2. Assembly

#### 2.2.1. Preparing components

**Fig 2:**
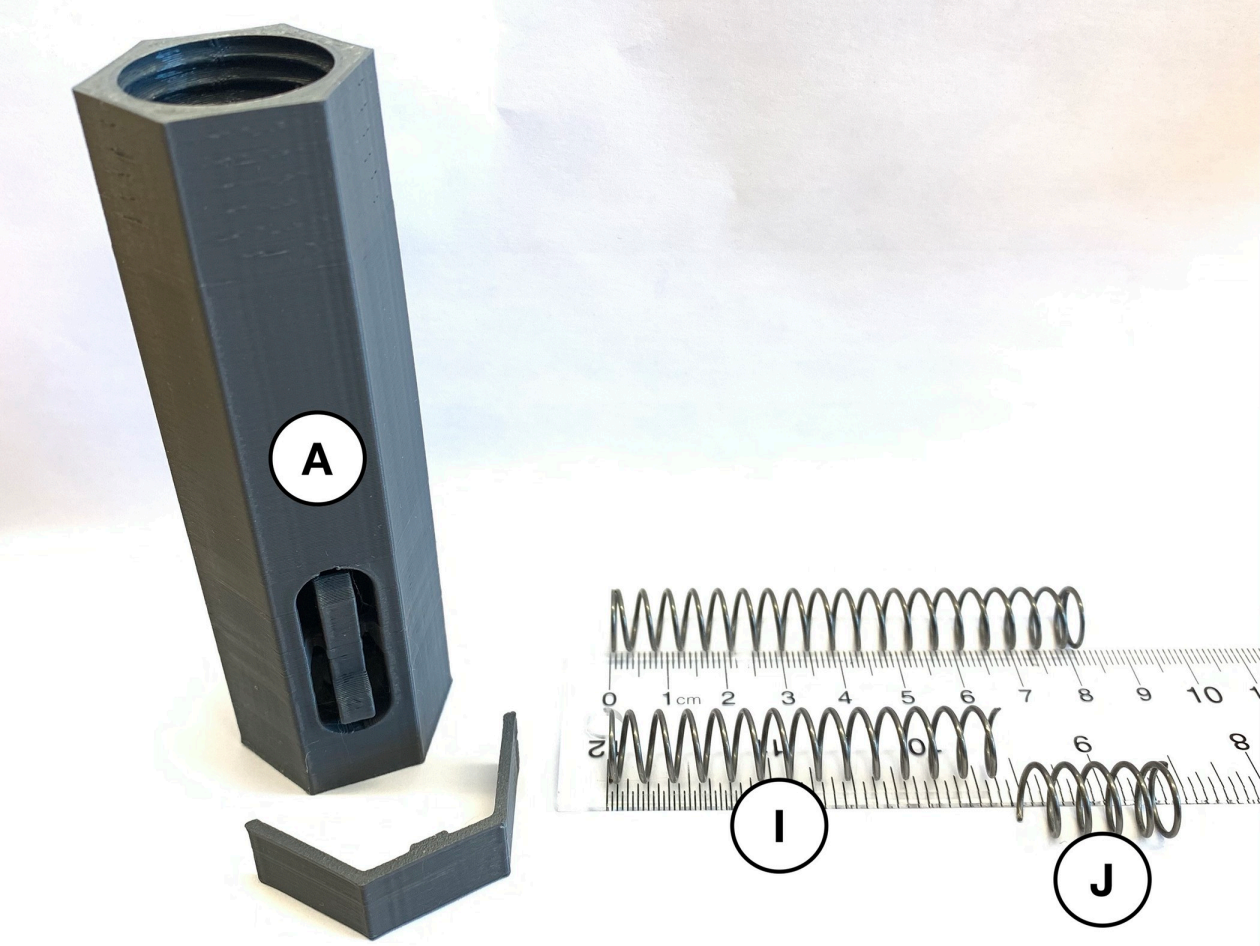
Remove built-in support on injector cover (*A*) and establish two springs (*I* and *J*).

Once all 3-D printed components are manufactured and the springs are purchased, the device is ready to be assembled. The grey injector cover (*A*) is printed with a built-in support which can be removed by separating the brace from the button. Use wire cutters on the 81.5 mm compression spring to produce a 61.5 mm or ~ ¾ fraction (*I*) and 20 mm or ~ ¼ fraction (*J*) of the full part. Place the injector cover on a flat surface with the threaded end facing up (Fig 2).

**Fig 3:**
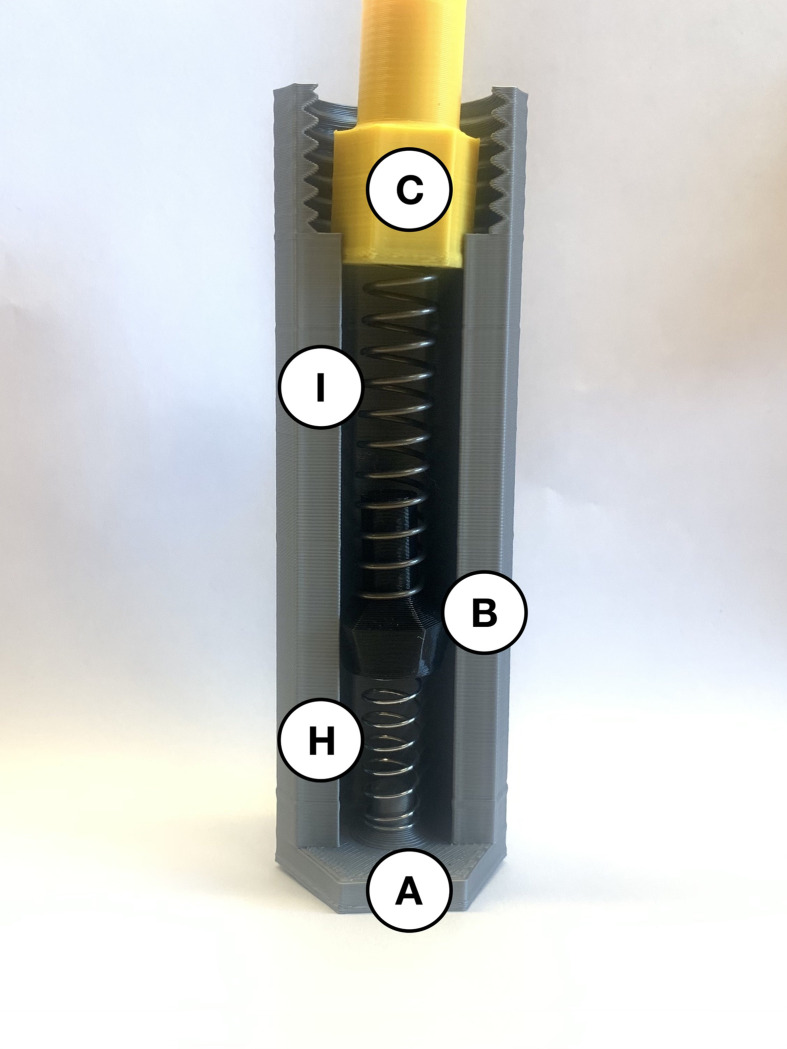
Internal view of the injector cover (*A*) assembly with parts *H*, *B*, *I*, and *C*.

To prepare the injection segment of the device, insert the 35 mm compression spring (*H*) so it is standing upright and in the centre. This spring is held in place by a cylindrical peg at the base of the injector cover (*A*). Place the black two-spring-hold (*B*) with the hole fitting around the 35 mm compression spring (*H*) and ensure they can compress and return to the initial position without any restrictions. Insert the 61.5 mm compression spring (*I*) so the inner edges encapsulate the cylindrical part of the black two-spring-hold (*B*). Place the yellow plunger (*C*) inside the injector cover (*A*) with the hole fitting around the larger compression spring (*I*). Thus, Fig 3 shows the 35 mm compression spring (*H*), two-spring-hold (*B*), the larger fraction of the 81.5 mm compression spring (*I*) and plunger (*C*) assembled within the injector cover (*A*) to create the necessary spring force.

**Fig 4:**
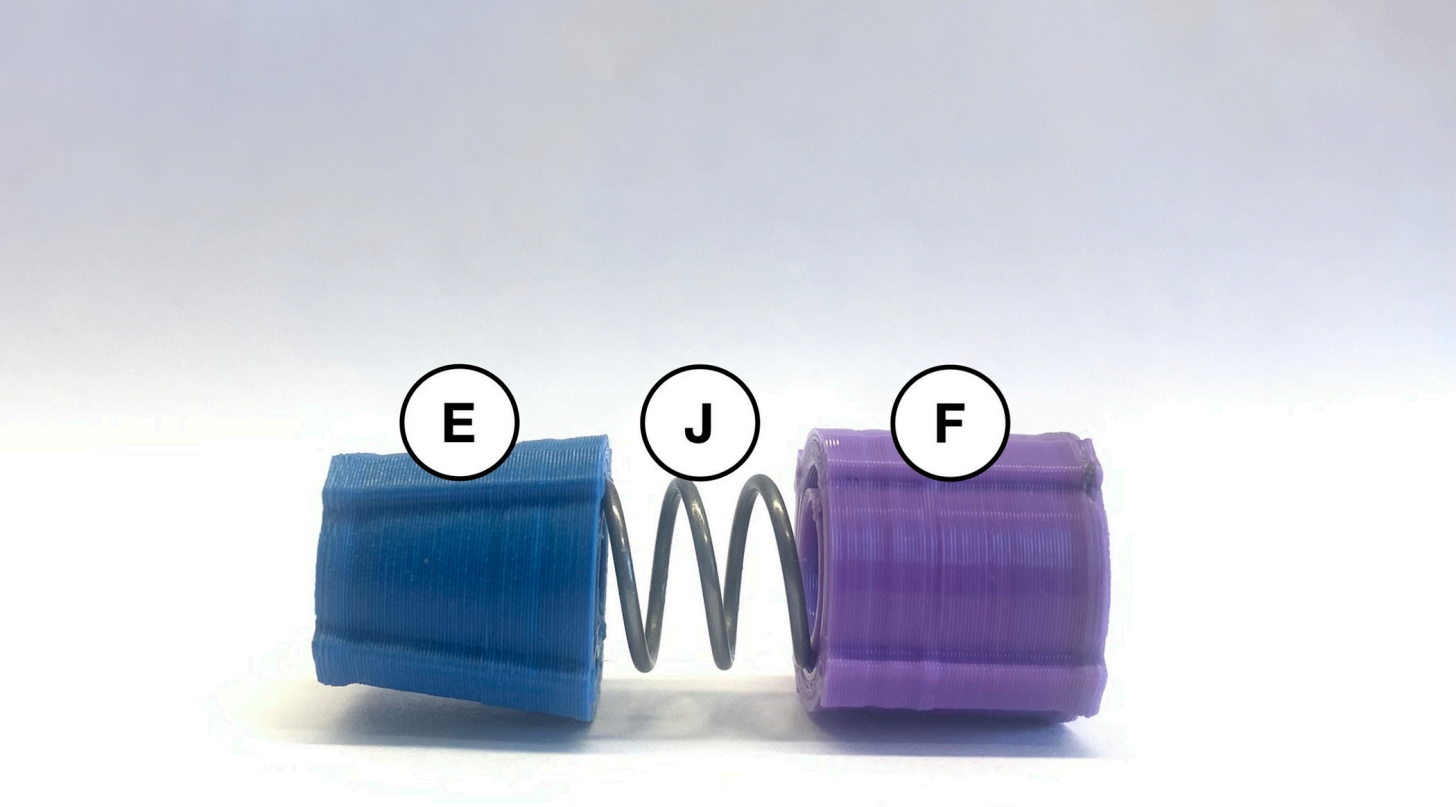
Blue (*E*) and purple (*F*) spring retainers fitted around the 20 mm compression spring (*J*).

**Fig 5:**
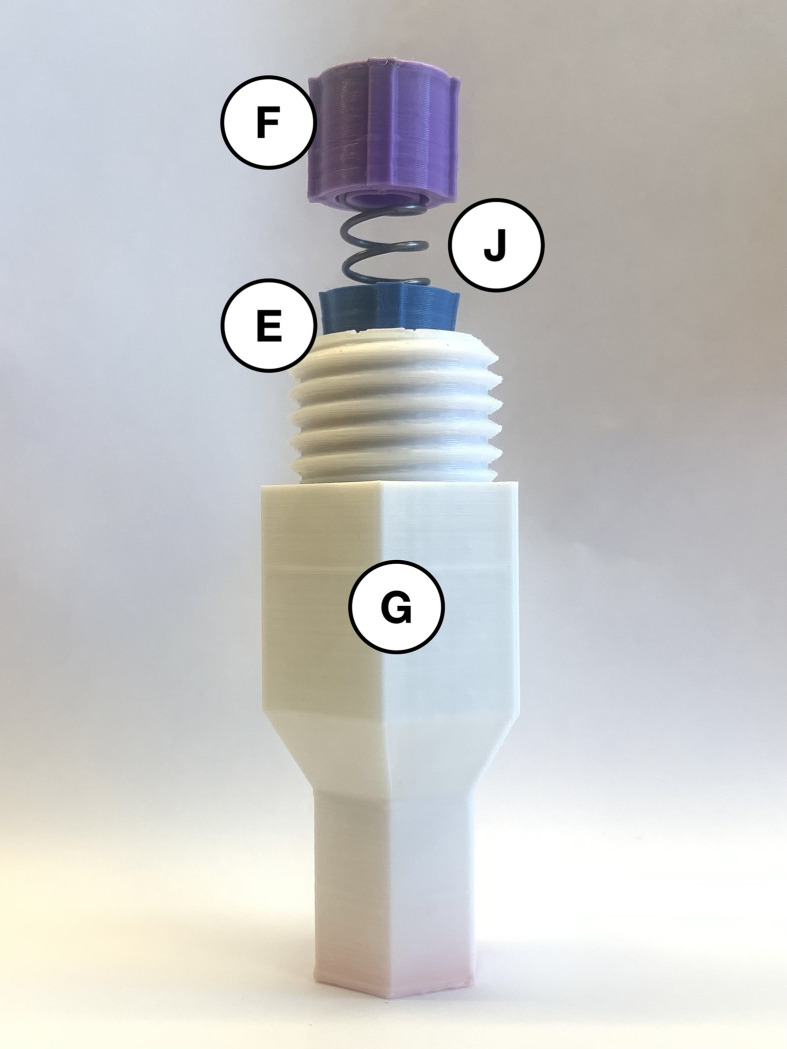
Syringe cover (*G*) prepared with parts *E*, *F*, and *J* to dampen the spring force.

When using the autoinjector, the 20 mm compression spring (*J*) is used inside the white syringe-cover (*G*) to dampen the spring force. As shown in Fig 4, the blue spring-cone-retainer (*E*) and purple spring-retainer (*F*) are attached to either end of the smaller spring (*J*) using the spring holes. Align the railings on both retainers (*E* and *F*) as much as possible by twisting them on the spring. Insert these assembled components with their railings fitted in the grooves of the larger opening of the syringe-cover (*G*). The correct orientation of the parts within the syringe segment of the device is shown in Fig 5. After the insulin syringe has been prepared and loaded into the device, the red connector (*D*) is used to connect the injection and syringe segments.

#### 2.2.2. Syringe safety

It should be noted that the red connector (D) performs two safety functions. First, the connector (*D*) is designed to provide a safety mechanism while loading the spring force (detailed in section 2.2.3 of the assembly). If the loaded spring force was prematurely released during the assembly process, the connector (*D*) would prevent the plunger (*C*) from shooting out of the injector cover (*A*) and potentially injuring the user. Secondly, connector (D) is redundant, and the device can be used without it, so it designed to have the thinnest and thus weakest points for breaking during dropping. Thus, if the device is dropped and broken connector D will be the part that breaks and can simply be printed and replaced if not an emergency. In the case of an emergency, it can be removed entirely (with the user being very careful not to prematurely release the device).

Before using the device, a medical professional must be consulted to determine the user’s appropriate syringe and needle length. The autoinjector was designed to be used with common insulin syringes that are prefilled with medication. Thus, a healthcare professional should be consulted to train the user on preparing their syringes and administering the medication. The following procedure for BD Insulin syringes detail how 100% ethyl alcohol was filled in each syringe (instead of insulin) during the autoinjector testing and validation [43]:

1. The procedure for BD Insulin syringes begins with the washing of hands and gathering supplies. To access the plunger, the white cap must be twisted and removed.
2. Use an alcohol swab to clean the top of the insulin bottle. If using cloudy insulin, the bottle should be gently rolled between the hands until it appears uniformly cloudy. The insulin bottle should not be shaken to prevent the formation of air bubbles.
3. To expose the needle, carefully twist off the orange shield without bending the needle or allowing it to touch anything.
4. Pull the syringe’s plunger down to the desired amount of units, making sure to leave an equal amount of air in the syringe as the amount of insulin prescribed.
5. Insert the needle through the center of the rubber top of the insulin bottle and push the plunger down fully.
6. Keep the needle in the bottle and carefully turn the bottle and syringe upside down, with the bottle on top.
7. Slowly pull the plunger down, aligning the thin black line of with the desired number of units on the syringe.
8. If air bubbles appear in the syringe, push the plunger up to inject the insulin back into vial, and then and redraw correct amount of insulin. Remove the syringe from bottle.
9. Check that the dose is correct amount of insulin, then clean a small, dry area of skin before injecting the insulin.

**Fig 6:**
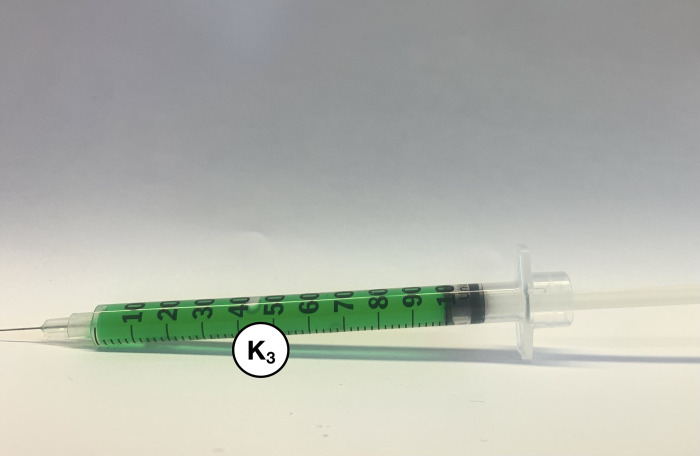
BD Insulin syringe (*K*_*3*_) loaded with 1.0 mL of ethyl alcohol and green food coloring.

The open-source autoinjector is compatible with a wide variety of syringes, and the Becton Dickenson (BD) insulin syringes used for testing are listed in Table 3. Six packs of ten syringes each were purchased from the University Hospital’s Prescription Centre at London Health Sciences Centre (LHSC) [44]. All used insulin syringes were safely disposed of in the appropriate sharps container. Fig 6 shows a 1.0 mL BD Insulin syringe with a 12.7 mm needle and 30 G (*K*_*3*_) filled with 100% ethyl alcohol and green food colouring. The OSF repository [29] includes a video of the full assembly of the device and the injection with the green-ethyl alcohol fluid.

**Table 3:** Tested Becton Dickinson (BD) insulin syringes with varying capacity, needle length, and gauge [45].

Part Number	Component	Capacity	Needle Length	Gauge	Cost (CAD$)
K_1_	10 BD Insulin Syringes with BD Ultra-Fine needle	1.0 mL	6 mm	31 G	7.49
K_2_	10 BD Insulin Syringes with BD Ultra-Fine needle	1.0 mL	8 mm	30 G	6.73
K_3_	10 BD Insulin Syringes with BD Ultra-Fine needle	1.0 mL	12.7 mm	30 G	7.20
K_4_	10 BD Insulin Syringes with BD Ultra-Fine needle	0.5 mL	8 mm	30 G	7.49
K_5_	10 BD Ultra-Fine Insulin Syringe	0.5 mL	12.7 mm	29 G	5.40
K_6_	10 BD Insulin Syringes with BD Ultra-Fine needle	0.3 mL	6 mm	31 G	6.99

#### 2.2.3. Loading device for injection

**Fig 7:**
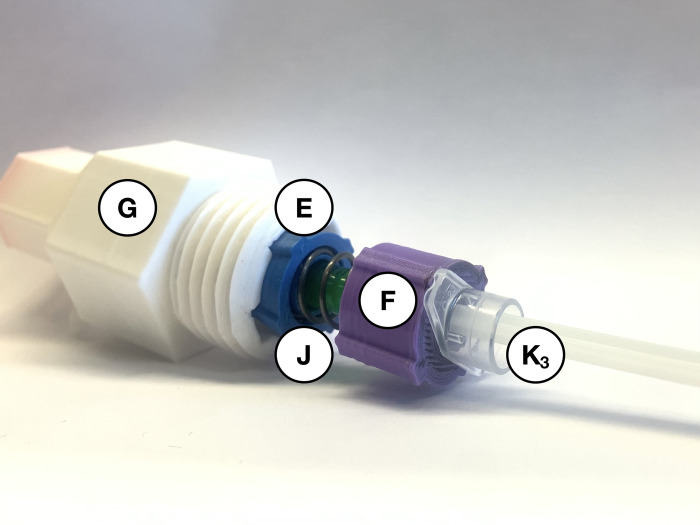
Align prefilled syringe (*K*_*3*_) with syringe cover (*G*), retainers (*E* and *F*), and small spring (*J*).

After assembling the device’s injection segment (Fig 3) and syringe segment (Fig 5), and one syringe from Table 3 is filled, the device is ready to be loaded and administered. Take the prefilled syringe (*K*_*3*_) and carefully thread the needle through the purple spring-retainer (*F*), the 20 mm compression spring (*J*) and then the blue spring-cone-retainer (*E*). Slowly assemble the syringe (*K*_*3*_) through the wider hole of the white syringe cover (*G*). Align the railings on the retainers (*E* and *F*) with the grooves inside the syringe cover (*G*) and turn the syringe (*K*_*3*_) to fit within the same indentations. Do not bend the needle, touch it with any components, or rest it against any surface. As shown in Fig 7, place the syringe cover (*G*) horizontally on a clean flat surface to load the spring mechanism in the injector cover (*A*).

**Fig 8:**
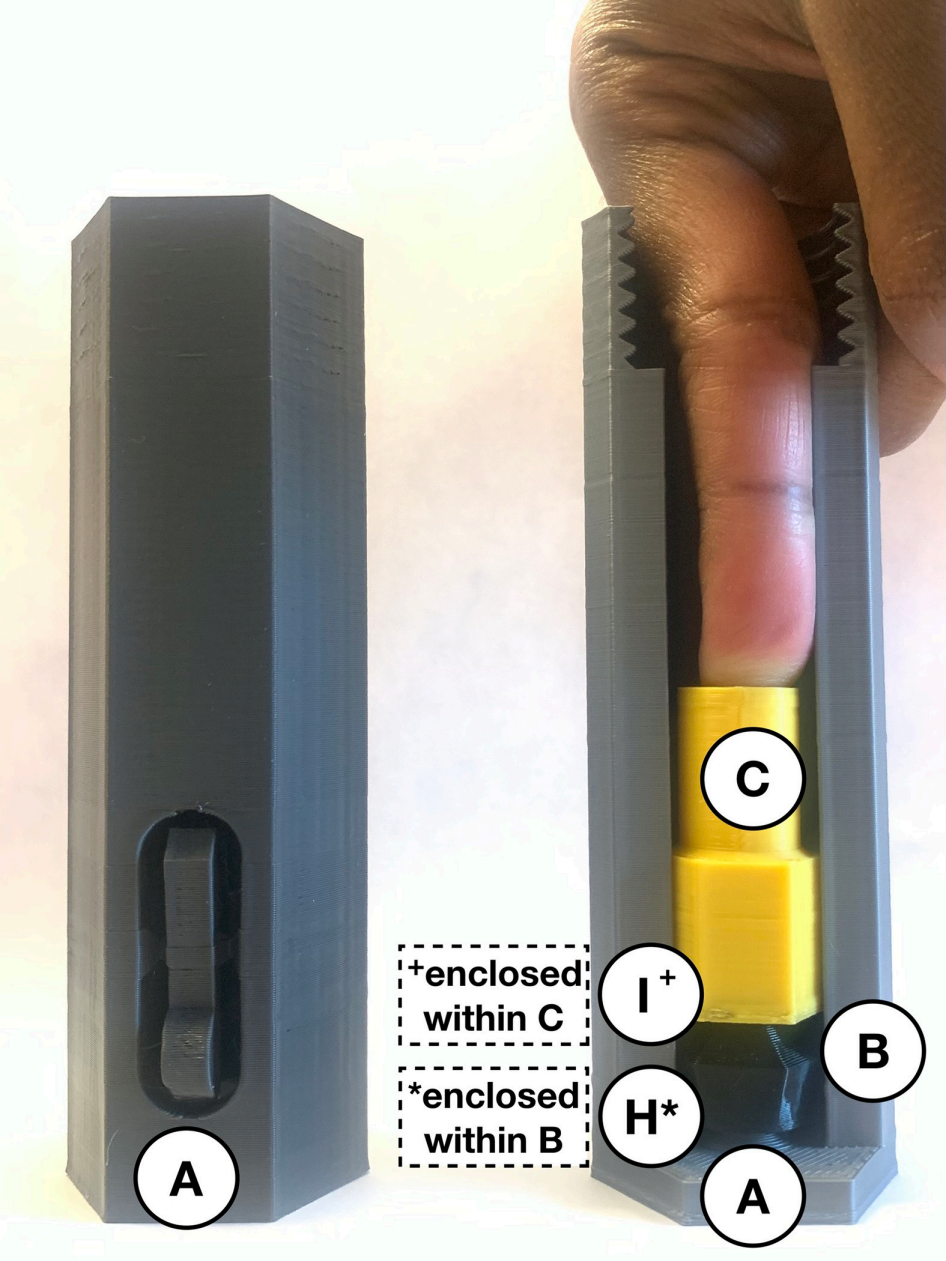
Pushing the plunger (*C*) inside the injector cover (*A*) to prepare the spring force.

**Fig 9:**
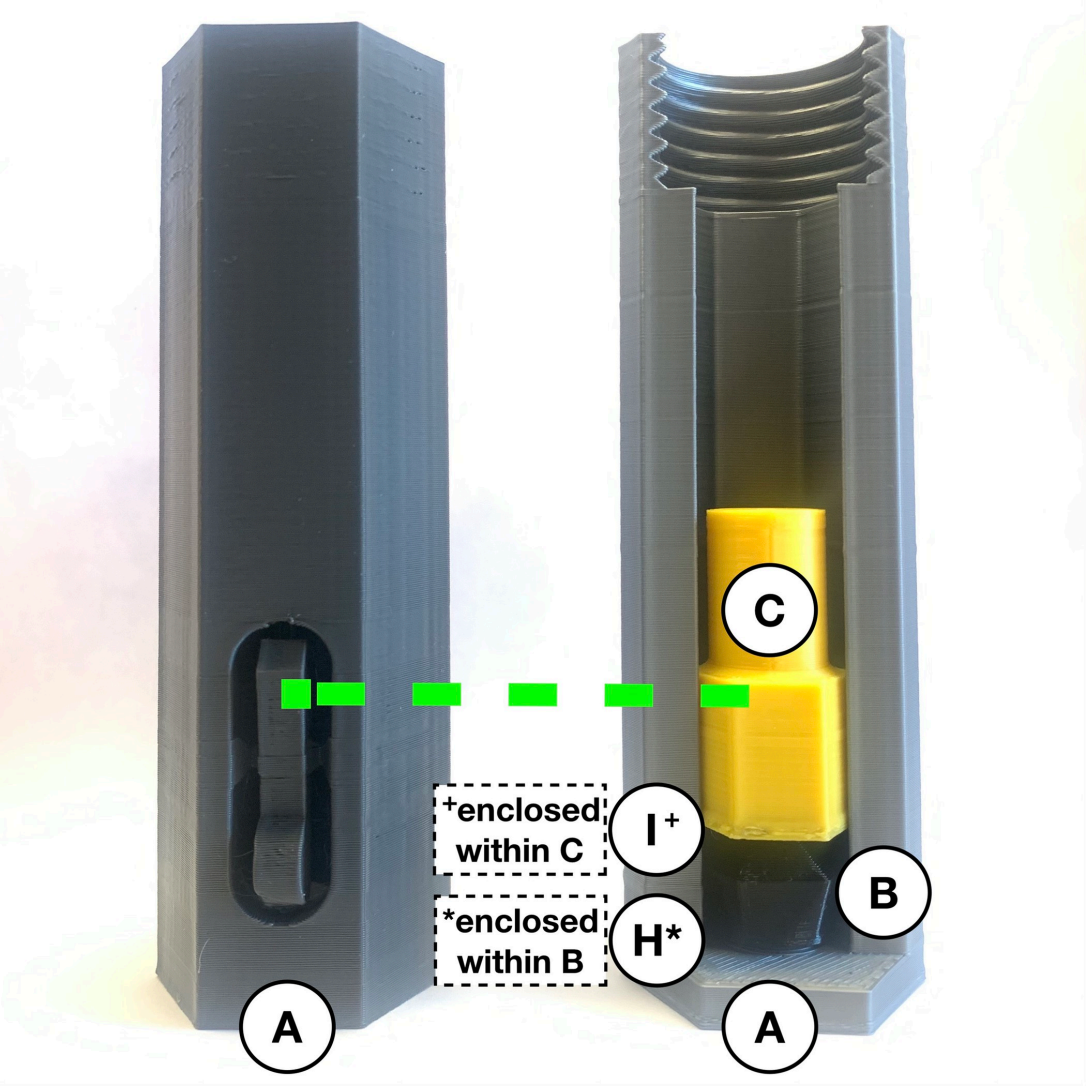
Distance that plunger (*C*) must be displaced to make contact and lock onto the button.

To prepare the spring-force, push down on the yellow plunger (*C*) with an index finger as shown in Fig 8 to compress the 35 mm (*H*) and 61.5 mm (*I*) compression springs. The spring force is locked at the base of the button of the injector cover (*A*), where it catches on the flat platform of the plunger (*C*). The button on the injector cover (*A*) is designed using a lever mechanism, where the loaded spring force is locked at one end of the button and released using the other part of the button. Fig 9 shows a horizontal green line between the base of the button and the vertical distance that the plunger (*C*) must be displaced to load the spring force. When the plunger (*C*) makes contact, it will be held down by the protruding ledge that is designed on the internal face of the button. Alternatively, the spring force can be released on the injector cover (*A*) when the circular end of the button is pushed, to bend the button away from the plunger (*C*). Thus, the locking mechanism is released, both compression springs (*H* and *I*) expand and push the plunger (*C*) toward the connector (*D*).

**Fig 10:**
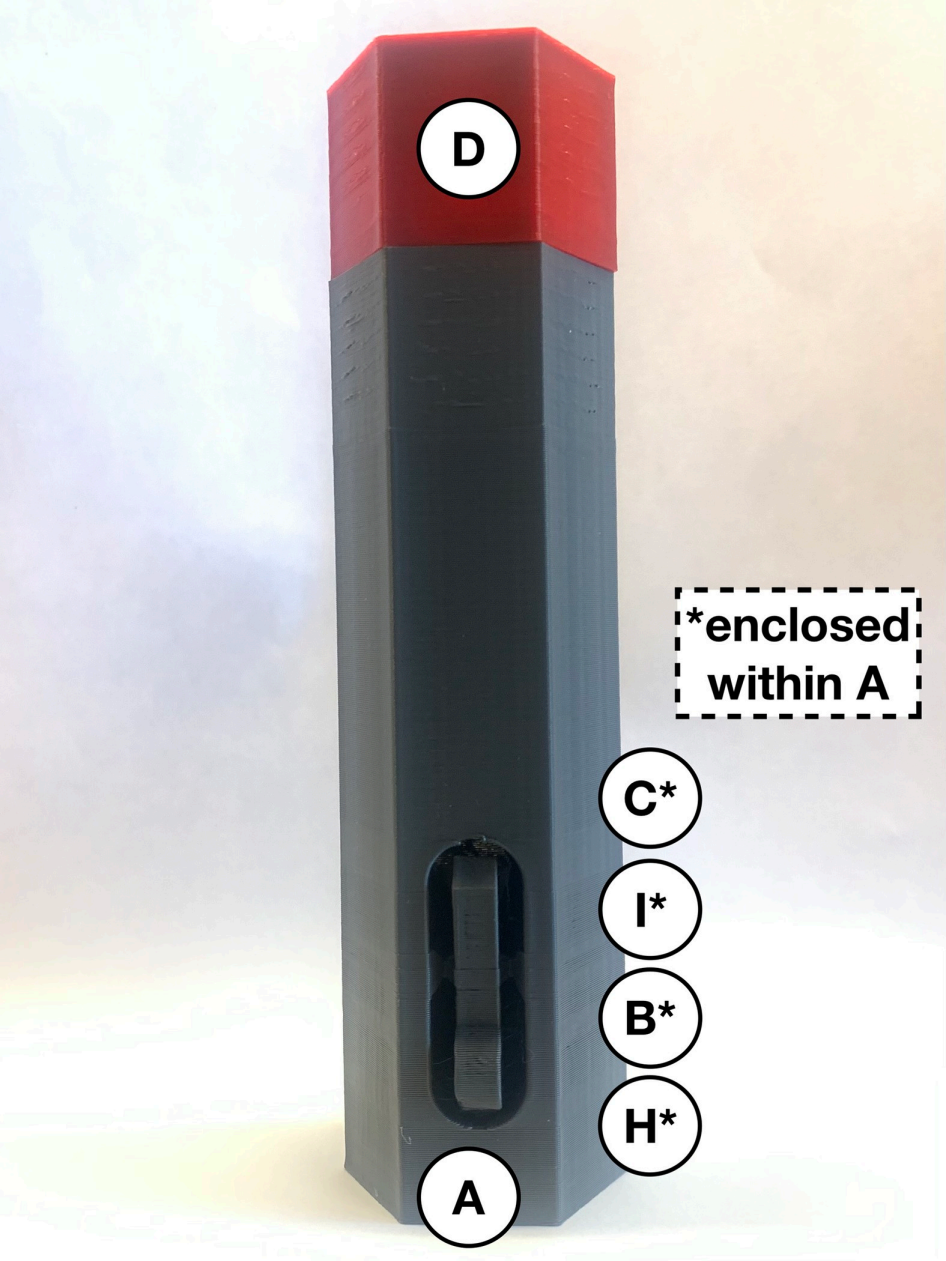
Loaded autoinjector (*A*, *H*, *B*, *I*, *C*, and *D*), ready for assembly with syringe component.

Load the spring force for the autoinjector by pushing the plunger (*C*) towards the button of the injector cover (*A*) until it locks. Once the spring force is locked, immediately screw the red connector (*D*) onto the injector cover (*A*) for safety (Fig 10). A locking mechanism is built into the connector (*D*) to contain parts *H*, *B*, *I*, and *C* within the injector cover (*A*), in case the loaded-springs were prematurely released. When the connector (*D*) is assembled with the injector cover (*A*), the six faces should be flush along both parts.

**Fig 11:**
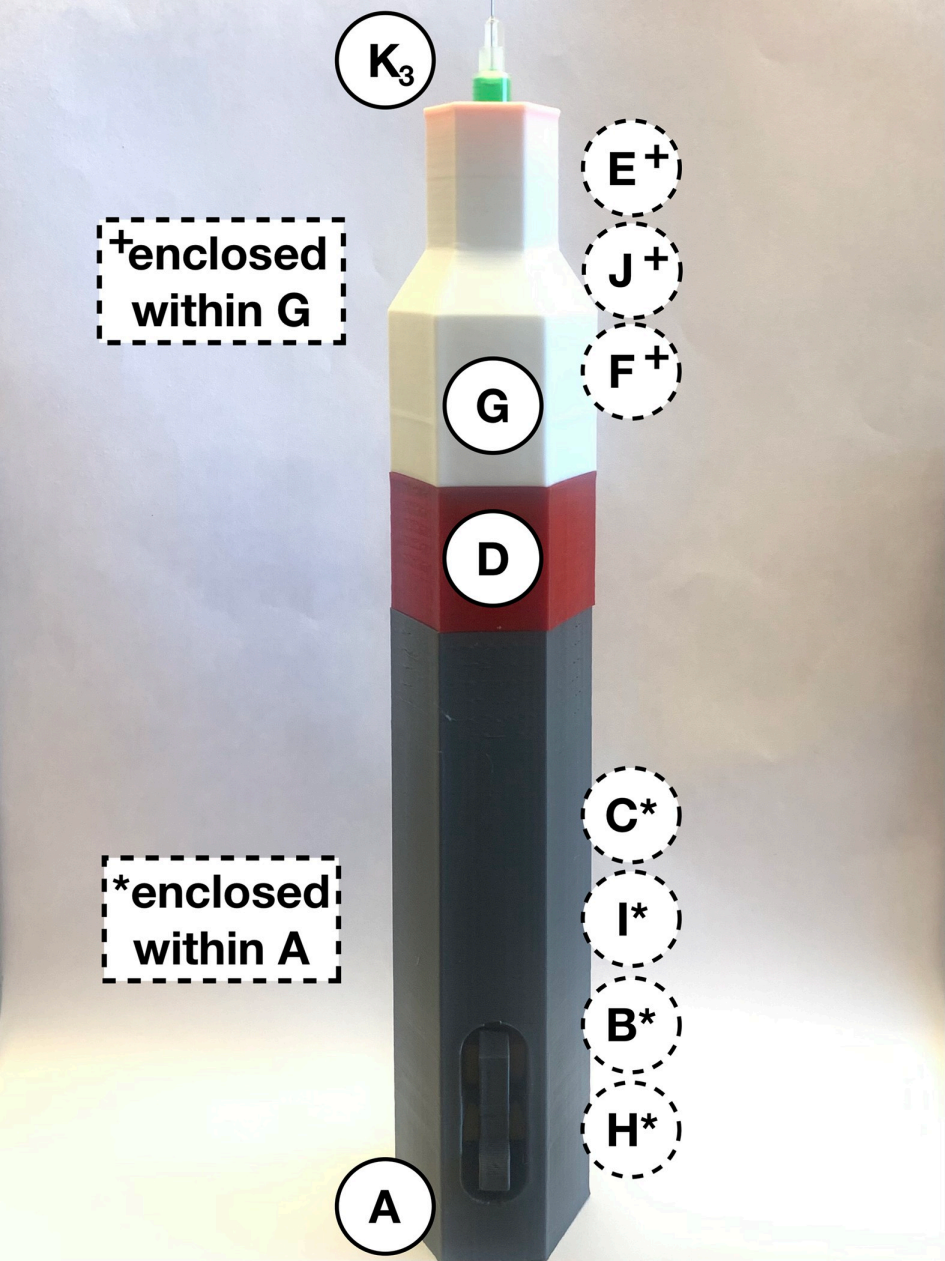
Fully assembled spring-driven autoinjector with a prefilled syringe, which is ready to be administered.

Take the loaded injection cover (*A*) with parts *H*, *B*, *I*, *C* and *D* and hold it with the connector aimed towards the ground in one hand. Carefully pick up the syringe cover (*G*) with parts *E*, *F*, *J*, and the prefilled syringe and hold it vertically in the other hand with the needle aimed towards the ground. Ensure the syringe is inserted straight, so the needle is not on an angle during the injection. Slowly move the insulin syringe to be lined up underneath the connector (*D*), then carefully move the syringe cover (*G*) closer. Without touching the syringe on the inside walls of the injector cover (*A*), vertically shift the syringe cover (*G*) until it can be screwed in with the connector (*D*). Attach the connector (*D*) with the syringe cover (*G*) and ensure both parts are flush with the injector cover (*A*) (Fig 11). The device is now assembled and the designated healthcare provider should be consulted about how to administer the injection from this point onwards. Hold the assembled autoinjector perpendicular to the injection area and release the medication by clicking the round button on the injector cover (*A*). Wait 10 seconds after administering the medication before carefully and slowly removing the needle from the injection area. Pull the device out at the same angle as the injection to avoid any injuries caused by the needle.

### 2.3. Experimental testing

#### 2.3.1. Validation

When using an autoinjector or any other injection device, administering the full dose of medication is vital. The open-source autoinjector was fabricated according to section 2.2, and then tested against the International Organization for Standardization (ISO 11608–1:2022 [30]) requirements for needle-based injection systems.

#### 2.3.2. Test liquid

Insulin was not used to test the autoinjector in an effort to avoid wasting the medicinal product, which would be more beneficial to diabetic patients. Thus, 100% ethyl alcohol was used as the test liquid because the viscosity is similar to that of insulin. Both fluids have a viscosity of approximately 1.1 mPa⋅s at 20°C [32, 33]. Additionally, the dose accuracy was tested by measuring the dose delivery efficiency for the full-capacity of syringes in Table 3 using 100% ethyl alcohol.

#### 2.3.3. Free-fall testing

Free-fall testing was used to assess the performance of the autoinjector after it was dropped onto a smooth surface of 3 mm thick steel from a height of 1 m. The height of 1 m was used to represent how far off the ground the autoinjector would be placed during preparation for use (such as on a tabletop). The 3 mm thick steel platform was rigidly attached to a wood backing whose thickness was greater than 10 mm. Before each device is dropped, the autoinjector was assembled with an insulin syringe (without removing the rigid needle shield (RNS)). The autoinjector was not dropped in a turbulent way.

According to ISO 11608–1, 20 devices should be dropped three times: once horizontally and twice vertically, rotating the device by 180° between the vertical drops. This test allows for a 15% failure rate to be considered passing and is developed for mass-scale manufacturing and thus is not entirely appropriate for this distributed manufacturing system. To reduce waste, only one autoinjector was used in the free-fall testing and it was dropped seven times in each orientation. For each orientation, the device could only be replaced once if broken in the seven drops (14.3% failure rate). Since one device was retested, the autoinjector was dropped horizontally, and vertically, then turned 180° and dropped vertically again before being dropped horizontally for the second time.

#### 2.3.4. Dose-delivery efficiency

The autoinjector is a needle-based injection system that uses a replaceable insulin syringe. Each syringe holds a single dose, and the device is designed to expel the entire deliverable volume. Therefore, the autoinjector is categorized under the system designation of B1 in ISO 11608–1. According to this standard, the accuracy of the device is evaluated as dose-delivery efficiency. As a user-filled single-dose system, the autoinjector was tested using six different BD insulin syringes (K_1-6_ from Table 3) that were filled to their full capacity. Five samples were collected for each of the six types of syringes for a total of 30 measurements. The dose-delivery efficiency percentage is calculated with the formula:

$$\frac{m_{2}-m_{3}}{m_{2}-m_{1}}\times 100$$ (1)

where:

*m*_1_ is mass of the device and container as received by the user (i.e. empty);

*m*_2_ is mass of the device and container as filled;

*m*_3_ is mass of the device and container and any residual volume after delivery.

A digital scale (Model: AB204-S, Mettler Toledo) with a repeatability of 0.1mg was used to determine m_1_, m_2_, and m_3_ for a total of 30 different syringes (n = 30). When determining the dose accuracy for the device, the *V*_set_ is defined as one of any pre-set doses expressed as a volume in millilitres. Thus, *V*_set1_ is equal to 100% capacity of the 1 mL syringes, *V*_set2_ is equal to 100% capacity of the 0.5 mL syringes and *V*_set3_ is equal to 100% capacity of the 0.3 mL syringes. Three different 1 mL syringes were tested (K_1-3_), for a total of 15 measurements in *V*_set1_. Two different 0.5 mL syringes were tested (K_4-5_), for the 10 measurements in *V*_set2_. Finally, there were five measurements made for K_6_ in *V*_set3_. Assuming that the dose efficiency measurements are normally distributed, the acceptance criteria would be that dose efficiency should fall within the 95% confidence intervals.

## 3. Results

### 3.1. Free-fall testing

Table 4 shows the results of the free fall test. When the device was dropped in the horizontal orientation, the connector (*D*) broke 3 out of 7 times. When dropped in vertical position with needle pointing up, the connector (*D*) broke once out of 7 times (round 6). In round 5, however, this component cracked, but was still usable. In round 6 it broke in the vertical (0°) drop. When dropped vertically with the needle pointing down (180°), the connector (*D*) failed twice out of 7 times. Throughout the entire free-fall testing, no other components broke after being dropped 21 times. Considering all 21 drops, the connector (*D*) broke at the joint of the thread and hexagonal prism for the 6 failed tests. Even though the failure rate is 28.5% (in all orientations), it should be noted that only the single safety part of the whole assembly was affected by the drop test. The rest of the components stayed perfectly intact. Moreover, the benefit of using 3-D printing as a method to manufacture the autoinjector is made apparent as wear-components might break or fail over time can be manufactured again with minimal cost and time.

**Table 4:** Outcomes of free-fall testing for one autoinjector that was dropped seven times in three orientations.

Round	Horizontal drop	Vertical (0°) drop (syringe pointing up)	Vertical (180°) drop (syringe pointing down)
1	Pass	Pass	Pass
2	Fail (connector)	Pass	Pass
3	Pass	Pass	Fail (connector)
4	Pass	Pass	Pass
5	Fail (connector)	Pass	Fail (connector cracked but was still useable for round 6)
6	Pass (connector remained usable)	Fail (connector broke)	Pass
7	Fail (connector)	Pass	Pass

### 3.2. Dose-delivery efficiency

Tables 5–7 show the dose-delivery efficiency for *V*_set1_, *V*_set2_, and *V*_set3_, respectively.

**Table 5:** Dose-delivery efficiency for *V*_*set1*_.

Insulin Syringe	Sample (n)	*m*_1_	*m*_2_	*m*_3_	Dose- efficiency
K_1_	1	80.0053	80.7947	80.0112	99.2526
	2	80.0083	80.8332	80.0102	99.7697
	3	79.9874	80.8153	80.0088	97.4151
	4	80.0455	80.8685	80.046	99.9392
	5	80.0438	80.8656	80.0476	99.5376
K_2_	6	80.0309	80.8337	80.0446	98.2935
	7	80.0437	80.8464	80.0486	99.3896
	8	79.8323	80.6486	79.836	99.5467
	9	79.8449	80.6606	79.8462	99.8406
	10	79.8614	80.6564	79.8663	99.3837
K_3_	11	79.9392	80.7642	79.9534	98.2788
	12	79.8725	80.6711	79.8746	99.7370
	13	79.8664	80.6555	79.8765	98.7201
	14	79.8666	80.6479	79.98	85.4857
	15	80.1931	80.9441	80.2232	95.9920

**Table 6:** Dose-delivery efficiency for *V*_*set2*_.

Insulin Syringe	Sample (n)	*m*_1_	*m*_2_	*m*_3_	Dose- efficiency
K_4_	16	80.8271	81.2165	80.8462	95.0950
	17	80.8317	81.2168	80.8408	97.6370
	18	80.7031	81.0849	80.7055	99.3714
	19	80.7045	81.1225	80.7114	98.3493
	20	80.7027	81.0818	80.7033	99.8417
K_5_	21	80.7166	81.1032	80.7217	98.6808
	22	80.7134	81.0945	80.7346	94.4372
	23	80.7121	81.0967	80.728	95.8658
	24	80.7654	81.1705	80.7706	98.7164
	25	80.9856	81.3774	80.9938	97.9071

**Table 7:** Dose-delivery efficiency for *V*_*set3*_.

Insulin Syringe	Sample (n)	*m*_1_	*m*_2_	*m*_3_	Dose- efficiency
K_6_	26	80.5808	80.7968	80.5902	95.6481
	27	80.5547	80.8004	80.5743	92.0228
	28	80.5041	80.7358	80.5413	83.9448
	29	80.5036	80.7435	80.5394	85.0771
	30	80.5051	80.7586	80.5112	97.5937

**Fig 12:**
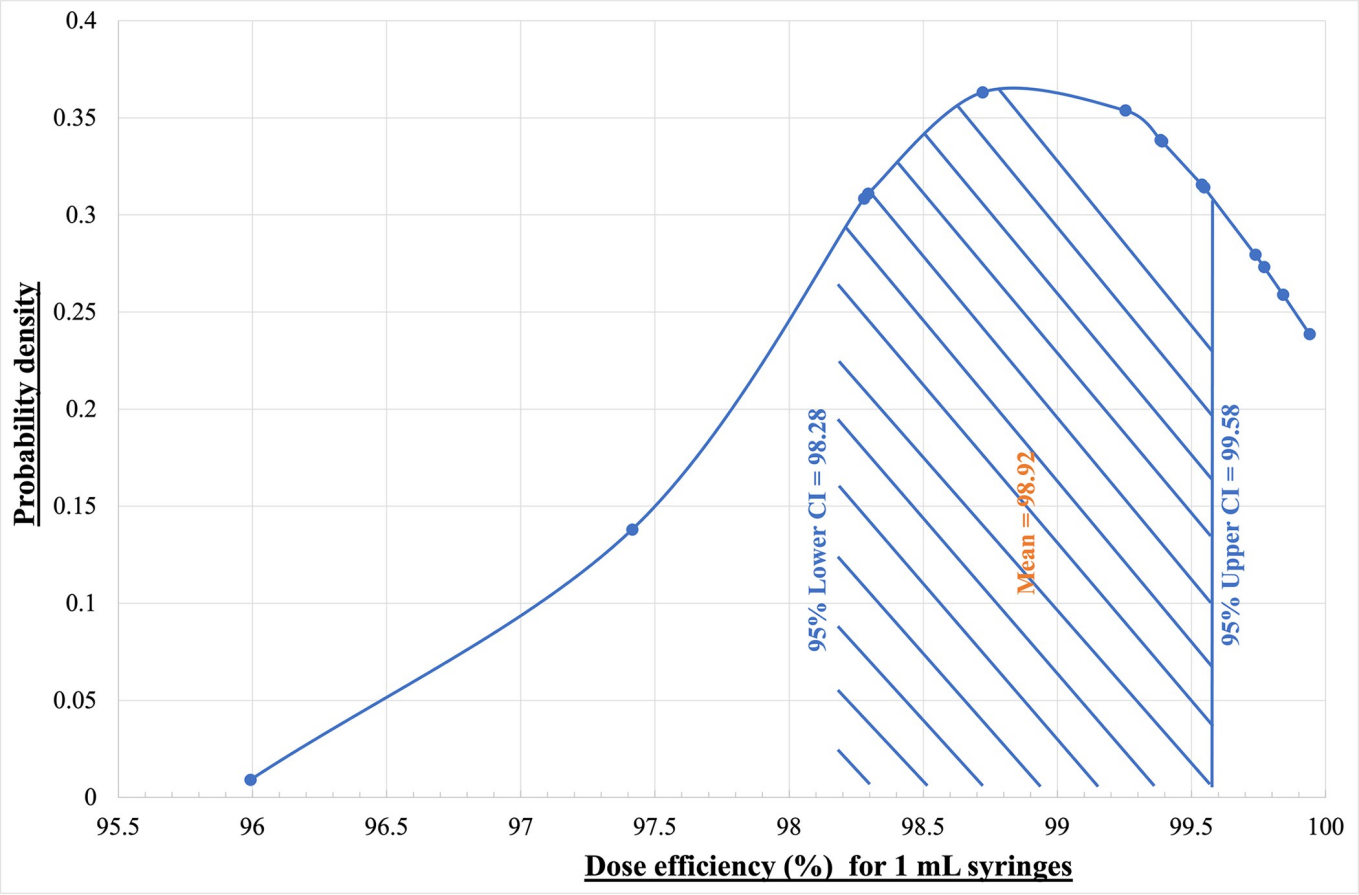
Gaussian distribution of the dose efficiency for the 1 mL BD Insulin syringes (*V*_*set1*_).

**Fig 13:**
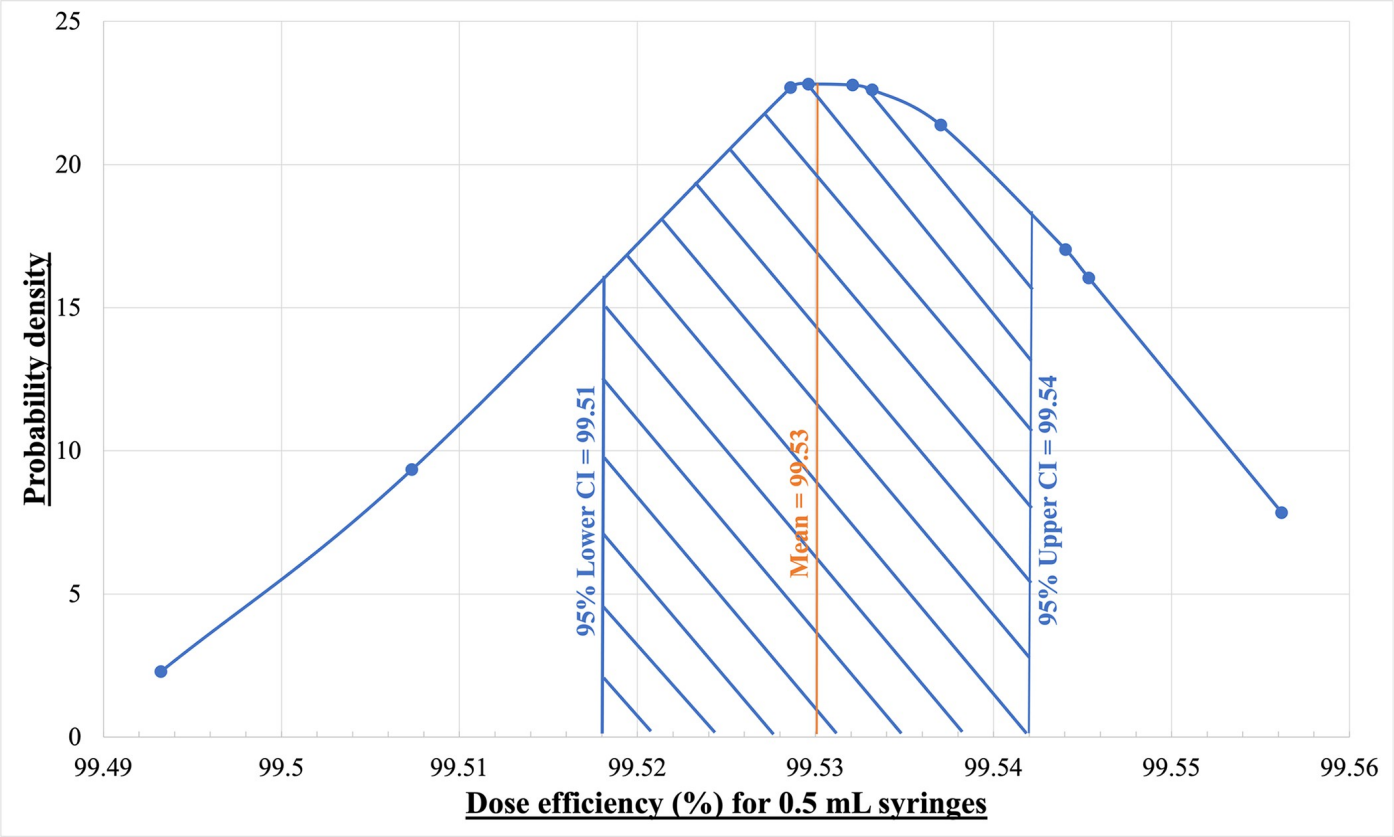
Gaussian distribution of the dose efficiency for the 0.5 mL BD Insulin syringes (*V*_*set2*_).

Fig 12 shows the normal distribution of the dose efficiency for the 1 mL syringes and Fig 13 shows the normal distribution of the dose efficiency for the 0.5 mL syringes. According to the statistical analysis, the population for both 1 mL and 0.5 mL syringes falls within a 95% confidence interval. For the 1 mL syringe, the upper level is 99.58% and the lower level is determined to be 98.28%. Therefore 95% of the time the dose efficiency of the 1 mL syringe will be within the upper and lower level of the confidence interval stated above. For the 0.5 mL syringes, the upper level of the 95% confidence interval is determined to be 99.54% and the lower level was 99.51%. Therefore 95% of the time the dose efficiency of the 0.5 mL syringe will fall between the upper and lower level of the confidence interval.

As can be seen in Table 7, injections using 0.3 mL syringes were not consistently successful because the autoinjector’s loaded spring force and size exceeded structural limitations of smaller syringes. Thus, this design cannot be used for 0.3 mL (or smaller) syringes without redesign.

## 4. Discussion

### 4.1. Validation of design

Upon following the validation procedures detailed in ISO 11608–1:2022 consistently had a dose-efficiency above 98% for 0.5 mL and 1.0 mL BD Insulin syringes. The design was able to function with 0.3 mL BD Insulin syringes, but the injections were not consistently successful. In comparison, 1.0 mL insulin syringes are more common and available for purchase. Thus, there was a small sample size for 0.3 mL syringes, which increased the variability of the dose-efficiency distribution. The autoinjector’s loaded spring force caused the syringe’s plunger (the plastic rod within the 0.3 mL BD Insulin syringe) to break in a few tests, affecting the efficiency of drug delivery. For 0.5 mL and 1 mL syringes, the syringes’ plunger and the device’s plunger (*C*) are within 15 mm of each other. There is a larger distance for 0.3 mL syringes, causing the released spring force to break the syringe’s plunger upon contact.

Future work could enable the use of the device with a 0.3 mL syringe, by shortening the injector cover (*A*) by approximately 30 mm. Therefore, the syringe’s plunger would make contact with the device’s plunger (*C*), and the autoinjector can be retested with the validation process detailed above. Future work should include developing a manual that includes the changes necessary to scale the autoinjector for syringes of different shapes and sizes beyond those tested here.

**Fig 14:**
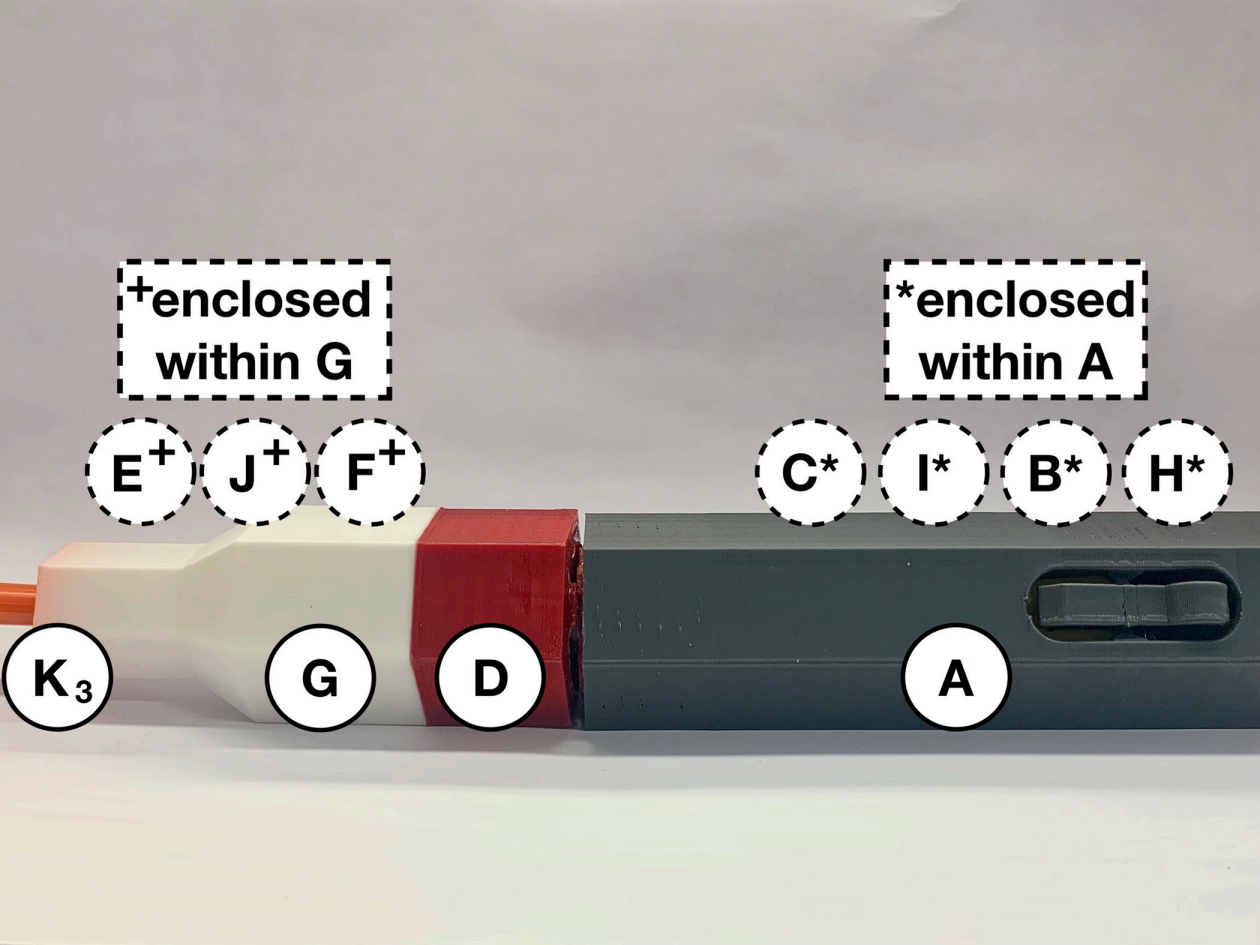
Broken connector (*D*) after dropping.

**Fig 15:**
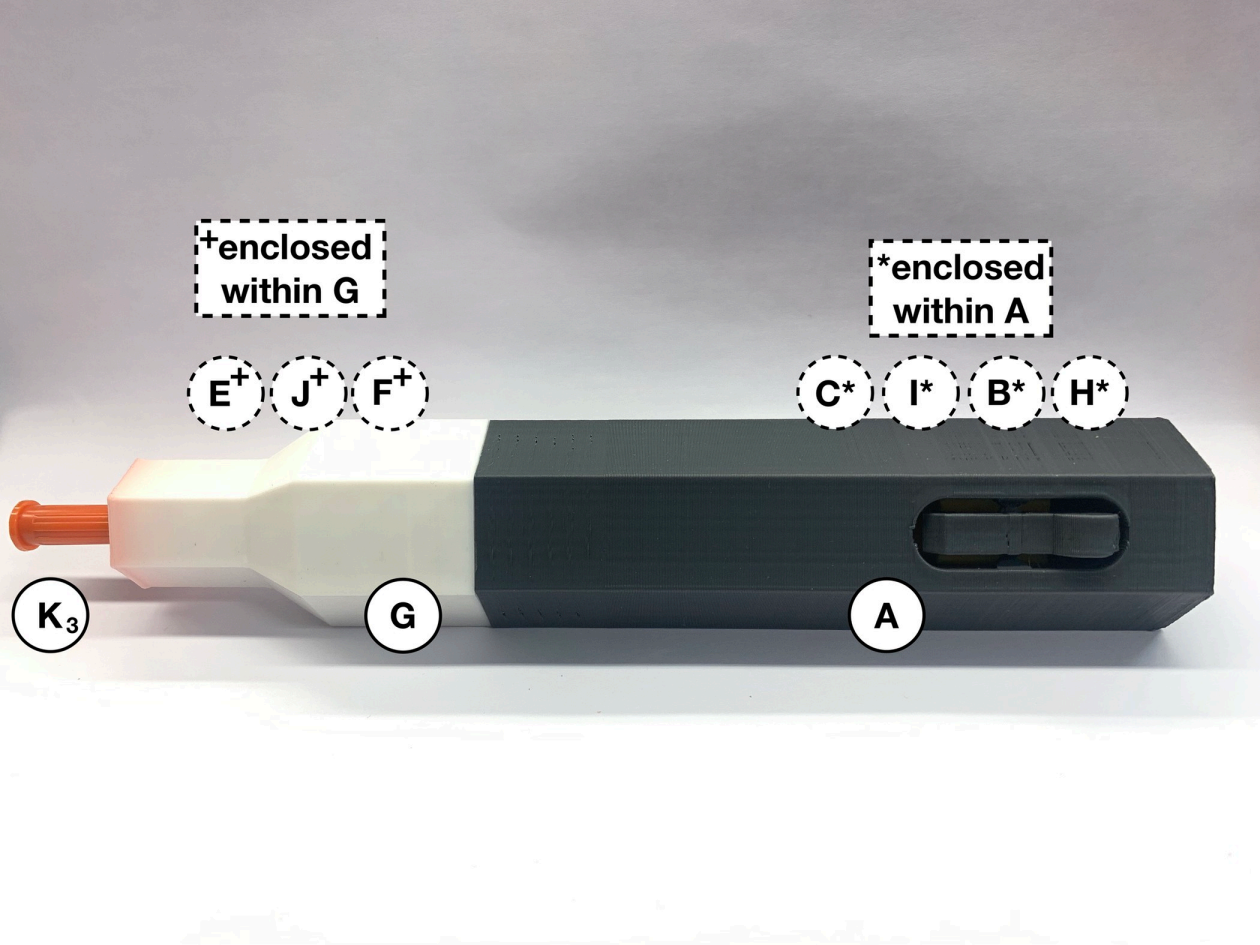
Open-source syringe pump assembled and able to function without connector (*D*).

As designed, during free-fall testing connector (*D*) cracked or visibly broke and required the part to be replaced. A representative example is shown in Fig 14. It should be made clear, the device can still be used without the connector (*D*), because the syringe cover (*G*) can screw directly into the injector cover (*A*). Thus, if necessary (e.g., in an emergency) the autoinjector can still be used without the connector (*D*) and this would not affect the functionality of the device (see Fig 15). From the free-fall testing, it is evident that if the device was to break, the connector is the most-likely component to be non-functional. Throughout this testing, it should also be noted that the syringe container was not damaged when the connector broke. Since the break in the connector (*D*) will be visible on the external face of the device, users will be able to remove the broken part prior to administering the medication. In this situation, the autoinjector can still be used without the broken connector (*D*), or until a new one can be re-printed in under 2 hours for CAD$0.29. For a design to pass the requirements detailed in the ISO 11608–1:2022, it must survive being dropped three times (once in each orientation) with up to 15% failure from 20 devices being tested. This design, however, instead failed a more rigorous protocol where one device was dropped seven times in each orientation. Therefore, the autoinjector can be considered more robust and valuable for users. This device can not only be modified to support the treatment of various health conditions, but in addition any damaged components can be quickly and affordably replaced instead of purchasing a new commercial autoinjector.

### 4.2. Ease of use of design

The autoinjector can be printed without supports and is thus relatively straight forward to manufacture with anyone with access to a RepRap-class FFF-based 3-D printer. The use of such printers for making biomedical equipment is now well established [45–47]. It is perhaps most mature for developing biomedical scientific tools such as microscopes for long-term live cell imaging [48–50], quartz crystal microbalances [51, 52], syringe pumps [53–57] to full automated liquid dispensing systems [58–60] and millifluidics [61]/microfluidics [62–64]. In general, open-source 3-D printed development of scientific tools saves scientists over 90% from purchasing equivalent equipment commercially [65] and many have begun to make open-source labs [66] made up of collections of customized open-source equipment [67]. This approach of distributed manufacturing is not only appropriate for scientists, as prosumers (producing consumers) are manufacturing adaptive aids for their own medical issues [68–70]. This device appears to fit within the scope of prosumers abilities that have experience with 3-D printing their own products. It is easy to both manufacture and assemble from a limited number of non-3-D printed components (2 springs). Similarly, the ease of use is similar to commercial autoinjectors, which generally have some form of method to secure the syringe inside the device and then has some form of trigger to use it as is done here. The open-source autoinjector can be used many times under normal use.

### 4.3. Cost savings

Commercial autoinjectors cost over CAD$100 [71, 72], while the open-source device tested here can be fabricated for less than CAD$7 in materials. These savings are consistent with the observations made about most 3-D printed products that prosumers are downloading to manufacture for themselves [73–76]. Savings over 90% means that in some jurisdictions (e.g., Ontario) the device can be manufactured for less than the sales tax on a proprietary device manufactured centrally and distributed at the retail scale. In addition, the ability to self manufacture opens up the potential to personalize the device (e.g., choose color or decorate with name, favorite sports team etc.) so that the device may have higher value to the prosumer [77, 78]. This ability to manufacture a superior device for less money challenges conventional business models [79] as well as supply chains [80–82]. The cost savings on a personal device are substantial enough that only printing a few autoinjectors would justify the cost of entry level desktop 3-D printer (e.g., the Ender 3 used in this study) would need about three injectors printed to recoup the capital investment in a weekend). The cost savings on an empty device are substantial and provide the opportunity for small local companies to have a profit stream while still reducing costs for their customers [83, 84].

### 4.4. Regulatory issues

Despite a clear technical path for lower cost devices, the difficulty, however, is that the U.S. Food and Drug Administration (FDA) and other similar regulatory organizations in other countries have rated autoinjectors as class II devices [85]. This class represents potential harm to patients and thus comes with substantial regulations to sell them. These regulations are currently not designed for distributed manufacturing (e.g., not only does the device need to be approved, but so does the manufacturing facility). This presents a challenge, but the new do-it-together (DIT) open hardware business model may be appropriate for this challenge [86–88]. In this model companies work together on an open hardware design (and in this case also FDA approval (or similar in other countries)) and then sell the products locally using peer production [89–91]. The regulatory oversight should be based on the nature of reasonably foreseeable adverse events. In general, if a 3-D printed device physical failure results in no patient damage or trivial foreseeable damage it should be considered very low risk. Similarly, even in cases where foreseeable damage could occur, if it is minor, and highly improbable it would also be considered very low risk.

When the autoinjector design failed during free-fall testing, the connector breaks did not result in the device being unable to function. Future regulatory work is needed to determine what risk having a non-functioning device represents for different situations. This device overcomes that risk, which is not observed in the current ISO standards or commercial devices. For this device, further testing is required to determine the conditions necessary for device non-function. Regulatory agencies should focus on the technical requirements (e.g., all administrative requirements represent barriers to entry for all non-traditional manufacturers, which do not make the products safer). Technical requirements for device function and safety should be strictly enforced using open and transparent standards so that all non-traditional manufacturers can provide the highest quality devices to the public at the lowest possible cost.

For a single small volume manufacturer, working with the FDA or other regulators to enable distributed manufacturing of class two devices is currently a major economic challenge and empty devices may not provide enough of a profit incentive for businesses to invest in regulatory compliance. In addition, to the team-like DIT strategy proposed above, there is an even greater profit potential for filled devices (e.g., Epipens that could justify the cost of a printer with even single autoinjector and purchased filled syringes with epinephrine). It appears, that the patent for the Epipen is still in force in the U.S. (it may not be in other locations). What is less clear is how the combination of a known drug and an autoinjector fulfilled the requirements of non-obviousness for patentability [92, 93] in the first place. In a similar fashion to how it is now obvious to all reasonably informed people that 3-D printing a known material is obvious [94]. This study should also make it clear to anyone that combining a known or future medication, drug or chemical with autoinjectors in general and the open-source autoinjector in this study in particular, is obvious and can not be patented.

As the open-source 3-D printable autoinjector is a class two medical device manufacturers must seek regulatory approval in their respective target market (e.g., FDA in the US, Health Canada in Canada [95], etc.). Although class 1 medical devices are now routinely developed to be made with distributed manufacturing (especially since the supply chain disruptions of the COVID-19 pandemic), class 2 and 3 devices are not. Substantial future work is needed to modernize the regulatory process to account for distributed manufacturing and enable devices that meet technical specifications to be produced and used. Currently, the system demands that facilities meet regulatory approval eliminates most entities that are not specifically dedicated to medical device manufacturing. One approach that might be applicable is to develop next generation monitoring of the manufacturing devices. For example, due to a flurry of recent work it is possible for 3-D printers can be monitored in real-time by leveraging machine learning to detect errors from layer-to-layer observation with cameras (computer vision) or laser scanning to ensure that there are no defects present [96–109]. If this process monitoring is coupled to a regulatory-approved design this may help provide a path to a regulatory approved new method of medical device manufacturing.

### 4.5. Risk analysis

The open-source 3-D printable autoinjector presents some potential risks that must be considered. One primary risk is the integrity of the 3-D printed components, as there is a possibility of weaknesses, defects or inconsistencies in the printed parts, which could lead to failure. It should be pointed out, however, the performed free-fall testing showed minimal failure probability of the critical safety components of the device that could cause harm. Safeguards to printing errors can include inspection of outer surface and computer vision monitoring during the print [103, 104].

Another risk to consider is the precision and accuracy of the injection dosage, as the 3-D printing process might introduce variations in the components affecting the consistency and the reliability. Inaccurate dosage delivery could lead to improper blood sugar management resulting in hypoglycemia or hyperglycemia. The precision and accuracy testing performed on this device, however, shows that the average accuracy is 98.9% for 1 mL syringes and 99.5% for 0.5 mL syringes, which is well above the acceptable standards according to ISO 11608–1.

According to ISO 14971:2019 [110] medical devices of this kind also involve risks associated with their intended use and reasonably foreseeable misuse. For example, the intended use of an autoinjector is for subcutaneous insulin injection by individuals with diabetes. Therefore, it is essential to consider the possibility of misuse, such as incorrect insertion of the syringe needle or improper handling of the device. Such misuse could result in unintended needle punctures, causing injury, infection, or tissue damage.

Several qualitative or quantitative characteristics could affect the safety of the medical device. Firstly, the sharpness and rigidity of the syringe needle play a crucial role in minimizing the risk of needle-related harm. If the needle is not sharp enough, it may cause pain, bruising, or difficulty penetrating the skin. Conversely, an excessively rigid needle may increase the likelihood of unintended penetration, leading to potential harm. For a needle-based injection system using prefilled syringes, the potential risks of the needle pertain to the insulin syringe selected, which should be assessed with the user’s physician. As stated in Section 2.2.2, a medical professional must be consulted to determine the user’s appropriate syringe and needle length before using the autoinjector. In addition, conducting usability studies and gathering feedback from users can help identify potential areas of risk and guide improvements in the device’s design and user interface. Quality control measures should also be implemented during manufacturing to ensure the sharpness, rigidity, and overall quality of the syringe needles. It should be pointed out here that the device tested uses commercial syringes and needles that have already gone through full regulatory approval.

The probability of the occurrence of harm and the severity and possible consequences must be carefully considered. The probability of harm occurring may be influenced by factors such as the user’s level of experience and training, their ability to follow instructions, and the design features of the autoinjector syringe. The severity of harm resulting from needle puncture injuries can range from minor discomfort and local irritation to more severe consequences, including infection, bleeding, or nerve damage.

To mitigate these risks, the design and construction of the autoinjector syringe should prioritize user safety. This may include incorporating features such as needle guards or safety mechanisms to minimize the risk of unintended needle punctures. A second version of components can be found in the OSF design repository [29], which improves the design of the syringe cover (part *G*). These changes allow 1 mL and 0.5 mL BD Insulin syringes to be assembled without removing the rigid needle shield. As a result, the needle and RNS are not exposed before medication is administered using the device. Additionally, clear and concise instructions for proper use should be provided to users, emphasizing correct needle insertion techniques and safe handling practices.

Furthermore, comprehensive user training programs and educational materials should be developed to enhance user understanding of the device and promote safe practices. By addressing these risks and implementing appropriate mitigation measures, the 3-D printed autoinjector syringe can minimize the probability of harm occurrence, reduce the severity of potential harm, and safeguard the well-being of users during insulin injection procedures.

### 4.6. Limitations and future work

The open-source 3-D printable autoinjector meets fluid delivery ISO-standards (ISO 11608–1:2022) for needle-based injection systems for 0.5 mL and 1 mL syringes. To use the design for smaller syringes, smaller springs and downsizing of the devices is needed. The device, which had a safety feature akin to a sheer pin added for free-falls, however, did not pass the free-fall testing as written. These free-fall tests, however, were not completely relevant to the devices manufactured in this way (e.g., printing dozens of autoinjectors) and for distributed manufactured devices having a redundant part act as a safety is a superior method for ensuring safety. There are several approaches to rectifying that challenge. First, the ISO standards should be modernized and revised to both account for distributed manufacturing and consider adding this feature to all autoinjectors, which for example could be sold with extra copies of the sacrificial safety component. If this not done and companies want to sell the open-source autoinjector, the system could also be redesigned. The same spot of the connector (*D*) broke each time at the base of the screw where it connects to the injector cover (*A*) (see Fig 14). It should be stressed, this redundant safety component is not necessary to operate the device as shown in Fig 15.

Other than the connector breaking as shown in Table 7, all the other components were functional after all 21 drops. which as noted earlier was far more drops than would normally be considered by the dropping of many devices a few times as governed by the current drop test ISO standard meant for mass manufactured devices. To ensure this design does not allow breaking of any component using the existing ISO standard, in future designs, the entire device (diameter of hexagon) could be enlarged to have more space to make those threads thicker without making the hexagon on the plunger smaller. The second, approach is simply to have the prosumer reprint the failed part if it is dropped and broken. Following the conventional system of mass-scale centralized manufacturing this is unthinkable, but following the new distributed manufacturing paradigm this approach makes far more sense. For example, having less bulky overly tough devices and components would cut down on manufacturing time and materials, which would decrease costs regardless of manufacturing context. With open-source designs that can simply be fixed by the prosumer when needed is more efficient. Far more work is needed in this general area of policy and law but is beyond the scope of this study. Another approach is to change the 3-D printed material. PLA, which was used here although the most common desktop 3-D printed plastic does not perform as well on impact tests as other relatively common 3-D printed plastics such as acrylonitrile butadiene styrene (ABS), glycol-modified polyethylene terephthalate (PETG), and polycarbonate (PC) materials [111, 112]. Future work could investigate this design with the ABS, PETG and PC.

## 5. Conclusions

This study successfully presented the design, manufacturing and testing against the current ISO standard (ISO 11608–1:2022) for needle-based injection systems of a spring-driven and 3-D printed autoinjector. The digitally replicable device is released under an open-source hardware license to provide a path to radically improve the accessibility of autoinjectors. The results show all of the components other than two springs can be 3-D printed in a little over 12 hours using an open-source desktop RepRap-class fused filament 3-D printer. The entire device bill of materials costs less than CAD$7 and thus decreases the cost of proprietary products by more than 90%. The safety and dosing accuracy was tested on a selection of commercial insulin syringes with varying capacities or needle lengths. Testing indicated that the entire dose was delivered over 97.5% of the time for 1 mL and 0.5 mL syringes. Injections using 0.3 mL syringes were not consistent and it is clear this design will need to be modified for 0.3 mL syringes. The new open-source design is more accessible to users than current commercial devices but should not be used now because it must be approved as it is a class two medical device. The autoinjector is a feasible and if future work can be done to enable regulatory approval for distributed 3-D printed class two medical devices then it can be used to support individuals burdened by healthcare costs. Regulation modernization is needed before it can be used if manufactured following a distributed manufacturing methodology.
